# ANXIETY IN COMMUNITY-DWELLING OLDER ADULTS AND ITS RELATED FACTORS DURING THE COVID-19 PANDEMIC

**DOI:** 10.1093/geroni/igac059.1802

**Published:** 2022-12-20

**Authors:** Yu Ma, Chieko Greiner, Hirochika Ryuno

**Affiliations:** Kobe University, Kobe, Hyogo, Japan; Kobe University, Kobe-city, Hyogo, Japan; Kobe University, Kobe city, Osaka, Japan

## Abstract

The rate of infection and magnitude of the effects of the coronavirus disease 2019 (COVID-19) pandemic has far exceeded the realm of what most people had previously experienced in their lives. There is an urgent need to ensure that strategies are in place to prevent and reduce anxiety in older adults. The present study aimed to assess anxiety in community-dwelling older adults and the related factors during the COVID-19 pandemic in Japan. A community-based cross-sectional study was completed in July 2021. The Japanese version of the Geriatric Anxiety Inventory was used to screen for anxiety symptoms. Demographical data and data for the following five other factors were collected: loneliness, mental well-being, perceived social support, coping behaviors to COVID-19, and fear of COVID-19. The association between anxiety and potential predictors was analyzed using binomial logistic regression. A total of 385 participants aged 65 to 86 years were included in the present study. 17% developed anxiety symptoms. Factors associated with anxiety were age, living alone, loneliness, significant people support, fear of COVID-19, and health monitoring as a COVID-19 coping behavior. Older age, living alone, and significant people support were associated with lower anxiety, and loneliness, fear of COVID-19, and health monitoring as a COVID-19 behavior were associated with higher anxiety in community-dwelling older adults during the COVID-19 pandemic. These findings can help to develop effective measures for reducing anxiety and will support the development of a psychological intervention for tackling the mental health of community-dwelling older adults during the COVID-19 pandemic.

